# Divergence Measures: Mathematical Foundations and Applications in Information-Theoretic and Statistical Problems

**DOI:** 10.3390/e24050712

**Published:** 2022-05-16

**Authors:** Igal Sason

**Affiliations:** 1 Andrew & Erna Viterbi Faculty of Electrical and Computer Engineering, Technion—Israel Institute of Technology, Haifa 3200003, Israel; eeigal@technion.ac.il; Tel.: +972-4-8294699; 2 Faculty of Mathematics, Technion—Israel Institute of Technology, Haifa 3200003, Israel

Data science, information theory, probability theory, statistical learning, statistical signal processing, and other related disciplines greatly benefit from non-negative measures of dissimilarity between pairs of probability measures. These are known as divergence measures, and exploring their mathematical foundations and diverse applications is of significant interest (see, e.g., [1,2,3,4,5,6,7,8,9,10] and references therein).

The present Special Issue, entitled *Divergence Measures: Mathematical Foundations and Applications in Information-Theoretic and Statistical Problems*, is focused on the study of the mathematical properties and applications of classical and generalized divergence measures from an information-theoretic perspective. It includes eight original contributions on the subject, which mainly deal with two key generalizations of the relative entropy: namely, the Rényi divergence and the important class of *f*-divergences. The Rényi divergence was introduced by Rényi as a generalization of relative entropy (relative entropy is a.k.a. the Kullback–Leibler divergence [11]), and it found numerous applications in information theory, statistics, and other related fields [12,13]. The notion of an *f*-divergence, which was independently introduced by Ali-Silvey [14], Csiszár [15,16,17], and Morimoto [18], is a useful generalization of some well-known divergence measures, retaining some of their major properties, including data-processing inequalities. It should be noted that, although the Rényi divergence of an arbitrary order is not an *f*-divergence, it is a one-to-one transformation of a subclass of *f*-divergences, so it inherits some of the key properties of *f*-divergences. We next describe the eight contributions in this Special Issue, and their relation to the literature.

Relative entropy is a well-known asymmetric and unbounded divergence measure [11], whereas the Jensen-Shannon divergence [19,20] (a.k.a. the capacitory discrimination [21]) is a bounded symmetrization of relative entropy, which does not require the pair of probability measures to have matching supports. It has the pleasing property that its square root is a distance metric, and it also belongs to the class of *f*-divergences. The latter implies, in particular, that the Jensen–Shannon divergence satisfies data-processing inequalities. The first paper in this Special Issue [22], authored by Nielsen, studies generalizations of the Jensen–Shannon divergence and the Jensen–Shannon centroid. The work in [22] further suggests an iterative algorithm for the numerical computation of the Jensen–Shannon-type centroids for a set of probability densities belonging to a mixture family in information geometry. This includes the case of calculating the Jensen–Shannon centroid of a set of categorical distributions or normalized histograms.

Many of Shannon’s information measures appear naturally in the context of horse gambling, when the gambler’s utility function is the expected log-wealth. The second paper [23], coauthored by Bleuler, Lapidoth, and Pfister, shows that, under a more general family of utility functions, gambling also provides a context for some of Rényi’s information measures. Motivated by a horse betting problem in the setting where the gambler has side information, a new conditional Rényi divergence is introduced in [23]. It is compared with the conditional Rényi divergences by Csiszár and Sibson, and the properties of all the three are studied in depth by the authors, with an emphasis on the behavior of these conditional divergence measures under data processing. In the same way that Csiszár’s and Sibson’s conditional divergences lead to the respective dependence measures, so does the new conditional divergence in [23] lead to the Lapidoth–Pfister mutual information. The authors further demonstrate that their new conditional divergence measure is also related to the Arimoto–Rényi conditional entropy and to Arimoto’s measure of dependence. In the second part of [23], the horse betting problem is analyzed where, instead of Kelly’s expected log-wealth criterion, a more general family of power-mean utility functions is considered. The key role in the analysis is played by the Rényi divergence, and the setting where the gambler has access to side information provides an operational meaning to the Lapidoth–Pfister mutual information. Finally, a universal strategy for independent and identically distributed races is presented in [23] which, without knowing the winning probabilities or the parameter of the utility function, asymptotically maximizes the gambler’s utility function.

The relative entropy [11] and the chi-squared divergence [24] are classical divergence measures which play a key role in information theory, statistical machine learning, signal processing, statistics, probability theory, and many other branches of mathematics. These divergence measures are fundamental in problems pertaining to source and channel coding, large deviations theory, tests of goodness-of-fit and independence in statistics, expectation–maximization iterative algorithms for estimating a distribution from an incomplete data, and other sorts of problems. They also belong to the generalized class of *f*-divergences. The third paper [25], by Nishiyama and Sason, studies integral relations between the relative entropy and chi-squared divergence, the implications of these relations, their information-theoretic applications, and some generalizations pertaining to the rich class of *f*-divergences. Applications that are studied in [25] include lossless compression, the method of types and large deviations, strong data-processing inequalities, bounds on contraction coefficients and maximal correlation, and the convergence rate to the stationarity of a type of discrete-time Markov chain.

The interesting interplay between inequalities and information theory has a rich history, with notable examples that include the relationship between the Brunn–Minkowski inequality and the entropy power inequality, transportation-cost inequalities and their tight connections to information theory, logarithmic Sobolev inequalities and the entropy method, inequalities for matrices obtained from the nonnegativity of relative entropy, connections between information inequalities and finite groups, combinatorics, and other fields of mathematics (see, e.g., [26,27,28,29,30]). The fourth paper by Reeves [31] considers applications of a two-moment inequality for the integral of fractional power of a function between zero and one. The first contribution of this paper provides an upper bound on the Rényi entropy of a random vector, expressed in terms of the two different moments. This also recovers some previous results based on maximum entropy distributions under a single moment constraint. The second contribution in [31] is a method for upper bounding mutual information in terms of certain integrals with respect to the variance of the conditional density.

Basic properties of an *f*-divergence are its non-negativity, convexity in the pair of probability measures, and the satisfiability of data-processing inequalities as a result of the convexity of the function *f* (and by the requirement that *f* vanishes at 1). These properties lead to *f*-divergence inequalities, and to information-theoretic applications (see, e.g., [4,10,32,33,34,35,36,37]). Furthermore, tightened (strong) data-processing inequalities for *f*-divergences have been of recent interest (see, e.g., [38,39,40,41,42]). The fifth paper [43], authored by Melbourne, is focused on the study of how stronger convexity properties of the function *f* imply improvements of classical *f*-divergence inequalities. It provides a systematic study of strongly convex divergences, and it quantifies how the convexity of a divergence generator *f* influences the behavior of the *f*-divergence. It proves that every (so-called) strongly convex divergence dominates the square of the total variation, which extends the classical bound provided by the chi-squared divergence. Its analysis also yields improvements of Bayes risk *f*-divergence inequalities, consequently achieving a sharpening of Pinsker’s inequality.

Divergences between probability measures are often used in statistics and data science in order to perform inference under models of various types. The corresponding methods extend the likelihood paradigm, and suggest inference in settings of minimum distance or minimum divergence, while allowing some tradeoff between efficiency and robustness. The sixth paper [44], authored by Broniatowski, considers a subclass of *f*-divergences, which contains most of the classical inferential tools, and which is indexed by a single scalar parameter. This class belongs to the family of *f*-divergences, and is usually referred to as the power divergence class, which has been considered by Cressie and Read [7,45]. The work in [44] states that the most commonly used minimum divergence estimators are maximum-likelihood estimators for suitably generalized bootstrapped sampling schemes. It also considers optimality of associated goodness-of-fit tests under such sampling schemes.

The seventh paper by Verdú [46] is a research and tutorial paper on error exponents and α-mutual information. Similarly to [23] (the second paper in this Special Issue), it relates to Rényi’s generalization of the relative entropy and mutual information. In light of the landmark paper by Shannon [47], it is well known that the analysis of the fundamental limits of noisy communication channels in the regime of vanishing error probability (by letting the blocklength of the code tend to infinity) leads to the introduction of the channel capacity as the maximal rate which enables to obtain reliable communication. The channel capacity is expressed in terms of a basic information measure: the input–output mutual information maximized over the input distribution. Furthermore, in the regime of fixed nonzero error probability, the asymptotic fundamental limit is a function of not only the channel capacity but the channel dispersion, which is expressible in terms of an information measure: the variance of the information density obtained with the capacity-achieving distribution [48]. In the regime of exponentially decreasing error probability, at fixed code rate below capacity, the analysis of the fundamental limits has gone through three distinct phases: (1) the early days of information theory and the error exponents analysis at MIT; (2) expressions for the error exponent functions by incorporating the relative entropy; and (3) the error exponent research with Rényi information measures. Thanks to Csiszár’s realization of the relevance of Rényi’s information measures to this problem [32], the third phase has found a way to express the error exponent functions as a function of generalized information measures, and also to solve the associated optimization problems in a systematic way. While in the absence of cost constraints, the problem reduces to finding the maximal α-mutual information, cost constraints make the problem significantly more challenging. The remained gaps in the interrelationships between three approaches, in the general case of cost-constrained encoding, motivated the present study in [46]. Furthermore, no systematic approach has been suggested so far for solving the attendant optimization problems by exploiting the specific structure of the information functions. The work by Verdú in [46] closes those gaps, while proposing a simple method to maximize the Augustin–Csiszár mutual information of order α under cost constraints [32,49], by means of the maximization of the α-mutual information subject to an exponential average constraint.

In statistical inference, the information-theoretic performance limits can often be expressed in terms of a statistical divergence measure between the underlying statistical models (see, e.g., [50] and references therein). As the data dimension grows, computing the statistics involved in decision making and the attendant performance limits (divergence measures) face complexity and stability challenges. Dimensionality reduction addresses these challenges at the expense of compromising performance because of the attendant loss of information. The eighth and last paper in the present Special Issue [51] considers linear dimensionality reduction, such that the divergence between the models is maximally preserved. Specifically, this work is focused on Gaussian models where discriminant analysis under several *f*-divergence measures are considered. The optimal design of the linear transformation of the data onto a lower-dimensional subspace is characterized for zero-mean Gaussian models, and numerical algorithms are employed to find the design for general Gaussian models with non-zero means.

It is our hope that the reader will find interest in the eight original contributions of this Special Issue, and that these works will stimulate further research in the study of the mathematical foundations and applications of divergence measures. 
